# Enhanced diagnostic and prognostic assessment of cardiac amyloidosis using combined ^11^C-PiB PET/CT and ^99m^Tc-DPD scintigraphy

**DOI:** 10.1007/s00259-025-07157-7

**Published:** 2025-02-28

**Authors:** Zhihui Hong, Clemens P. Spielvogel, Song Xue, Raffaella Calabretta, Zewen Jiang, Josef Yu, Kilian Kluge, David Haberl, Christian Nitsche, Stefan Grünert, Marcus Hacker, Xiang Li

**Affiliations:** 1 Department of Nuclear Medicine, The Second Affiliated Hospital of Soochow University, Suzhou, 215002 China; 2 Division of Nuclear Medicine, Department of Biomedical Imaging and Image-guided Therapy, Vienna General Hospital, Medical University of Vienna, Währinger Gürtel 18-20, Floor 3L, Vienna, 1090 Austria; 3 Division of Cardiology, Department of Internal Medicine II, Medical University of Vienna, Vienna, Austria; 4 Christian Doppler Laboratory for Applied Metabolomics, Medical University of Vienna, Vienna, Austria; 5 Department of Nuclear Medicine, Beijing Chest Hospital, Capital Medical University, Beijing, China

**Keywords:** Cardiac amyloidosis, ^11^C-PIB PET/CT, ^99m^Tc-DPD scintigraphy, Subtype differentiation, Clinical prognosis

## Abstract

**Background:**

Cardiac amyloidosis (CA) is a severe condition characterized by amyloid fibril deposition in the myocardium, leading to restrictive cardiomyopathy and heart failure. Differentiating between amyloidosis subtypes is crucial due to distinct treatment strategies. The individual conventional diagnostic methods lack the accuracy needed for effective subtype identification. This study aimed to evaluate the efficacy of combining ^11^C-PiB PET/CT and ^99m^Tc-DPD scintigraphy in detecting CA and distinguishing between its main subtypes, light chain (AL) and transthyretin (ATTR) amyloidosis while assessing the association of imaging findings with patient prognosis.

**Methods:**

We retrospectively evaluated the diagnostic efficacy of combining ^11^C-PiB PET/CT and ^99m^Tc-DPD scintigraphy in a cohort of 50 patients with clinical suspicion of CA. Semi-quantitative imaging markers were extracted from the images. Diagnostic performance was calculated against biopsy results or genetic testing. Both machine learning models and a rationale-based model were developed to detect CA and classify subtypes. Survival prediction over five years was assessed using a random survival forest model. Prognostic value was assessed using Kaplan-Meier estimators and Cox proportional hazards models.

**Results:**

The combined imaging approach significantly improved diagnostic accuracy, with ^11^C-PiB PET and ^99m^Tc-DPD scintigraphy showing complementary strengths in detecting AL and ATTR, respectively. The machine learning model achieved an AUC of 0.94 (95% CI 0.93–0.95) for CA subtype differentiation, while the rationale-based model demonstrated strong diagnostic ability with AUCs of 0.95 (95% CI 0.88-1.00) for ATTR and 0.88 (95% CI 0.770–0.961) for AL. Survival prediction models identified key prognostic markers, with significant stratification of overall mortality based on predicted survival (*p* value = 0.006; adj HR 2.43 [95% CI 1.03–5.71]).

**Conclusion:**

The integration of ^11^C-PiB PET/CT and ^99m^Tc-DPD scintigraphy, supported by both machine learning and rationale-based models, enhances the diagnostic accuracy and prognostic assessment of cardiac amyloidosis, with significant implications for clinical practice.

**Graphical abstracts:**

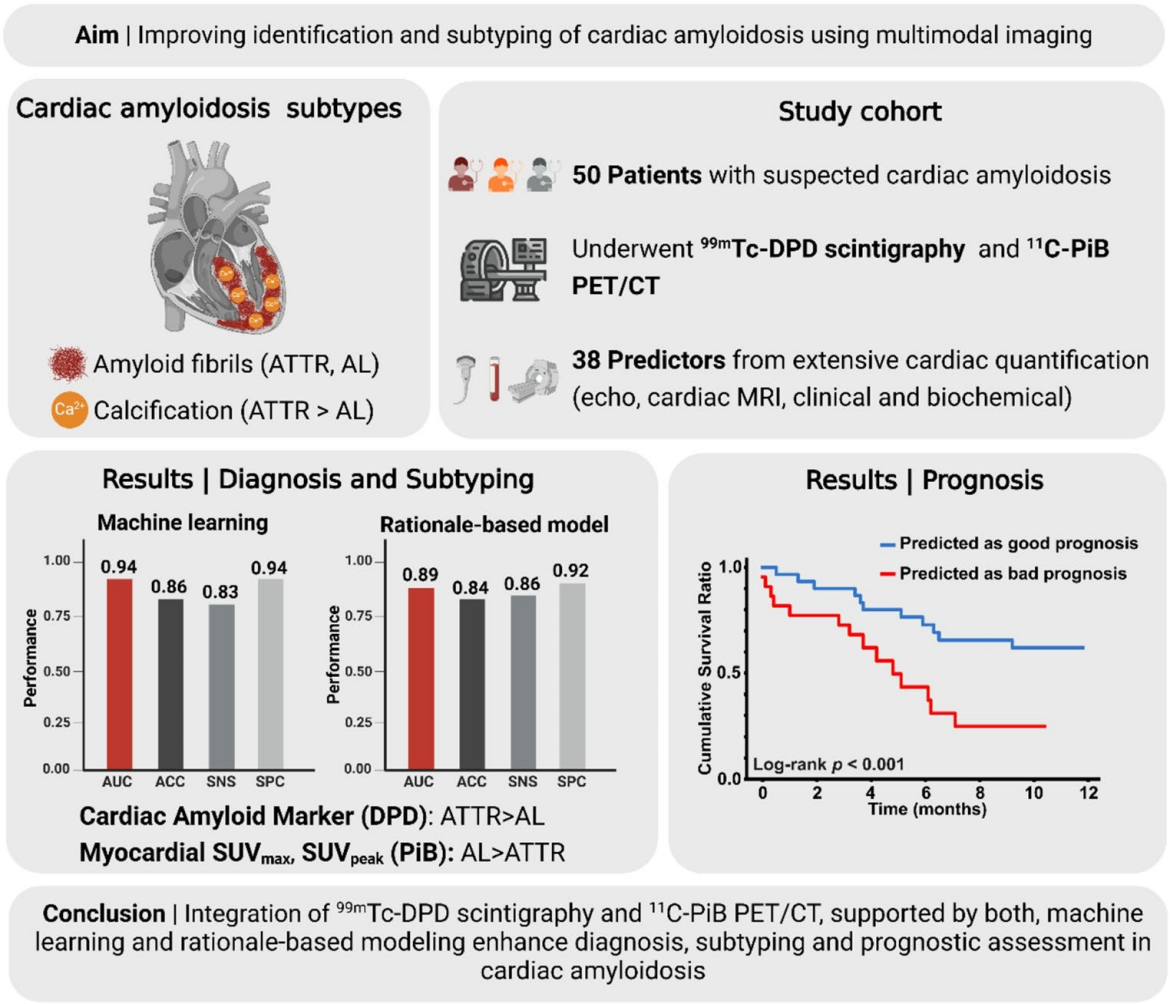

**Supplementary Information:**

The online version contains supplementary material available at 10.1007/s00259-025-07157-7.

## Introduction

Cardiac amyloidosis (CA) is a life-threatening cardiac disease characterized by the extracellular deposition of amyloid fibrils within the myocardium, ultimately leading to restrictive cardiomyopathy and heart failure. The two principal forms of CA are light-chain amyloidosis (AL) and transthyretin amyloidosis (ATTR). AL amyloidosis results from the deposition of immunoglobulin light chains produced by clonal plasma cells, whereas ATTR amyloidosis is caused by the misfolding of transthyretin protein, which can occur in either its wild-type form (wtATTR) or hereditary-type form (hATTR).

Differentiating between AL and ATTR amyloidosis is critical due to their distinct therapeutic and prognostic implications. AL amyloidosis often necessitates immediate and aggressive chemotherapy to suppress the underlying plasma cell disorder [1, 2], while the management of ATTR amyloidosis may involve TTR stabilizers, gene-silencing therapies, or organ transplantation, particularly in advanced cases [3–5]. Accurate and timely diagnosis is essential to initiate appropriate treatment and improve patient outcomes.

Traditional diagnostic approaches, such as echocardiography, cardiac magnetic resonance imaging (MRI), and endomyocardial biopsy, have been instrumental in detecting and characterizing CA. However, these techniques face limitations in their ability to reliably distinguish between AL and ATTR subtypes. Echocardiography and MRI provide structural and functional information about the heart but lack specificity in identifying amyloid subtypes. Endomyocardial biopsy, while definitive, is invasive and carries procedural risks.

Advances in radionuclide imaging have introduced new modalities that offer enhanced specificity for amyloid subtypes. ^11^C-PiB (carbon-11 labelled Pittsburgh compound B) positron emission tomography (PET) and ^99m^Tc-DPD (technetium-99m-labelled 3,3-diphosphono-1,2-propanodicarboxylic acid) scintigraphy are two conventional techniques employed in clinical practice. ^11^C-PiB PET, initially developed for imaging amyloid plaques in Alzheimer’s disease, has shown promise in visualizing amyloid deposits within the myocardium [6]. This imaging modality leverages the affinity of ^11^C-PiB for amyloid fibrils, enabling the detection of cardiac amyloid burden. In contrast, ^99m^Tc-DPD scintigraphy exhibits high sensitivity and specificity for ATTR amyloidosis due to its preferential binding to transthyretin fibrils, making it a valuable tool for diagnosing this subtype.

Given the complementary information of these imaging modalities, combining ^11^C-PiB PET and ^99m^Tc-DPD scintigraphy may provide a more comprehensive and accurate diagnostic approach for differentiating AL and ATTR CA. Furthermore, the extent of amyloid deposition detected by these imaging techniques could potentially correlate with clinical outcomes, particularly survival, thus serving as prognostic indicators.

The primary aim of this study was to evaluate the efficacy of combining ^11^C-PiB PET and ^99m^Tc-DPD scintigraphy in detecting CA and distinguishing AL from ATTR subtypes. Additionally, we seek to explore the correlation between imaging findings and patient prognosis, focusing on survival outcomes. By integrating these advanced imaging techniques, we hypothesized that a combined diagnostic approach would enhance the accuracy of subtype differentiation and provide valuable prognostic information, ultimately informing clinical decision-making and improving patient management.

## Methods

### Patients

We retrospectively included patients with clinical suspicion of CA based on symptoms, echocardiographic findings, and/or cardiac MRI results suggestive of amyloid infiltration at Vienna General Hospital between 2014 and 2021. The inclusion criteria were: Clinical presentation consistent with CA (e.g., heart failure symptoms, thickened ventricular walls on echocardiography, characteristic late gadolinium enhancement on MRI), or biopsy-confirmed diagnosis of either ATTR-CA or AL-CA. The duration between ^99m^Tc-DPD scintigraphy and ^11^C-PiB PET was no longer than 1 year for any patient. Exclusion criteria were: patients lacking biopsy-proven diagnosis.

Patient demographics, clinical history, laboratory results (including serum-free light chains, proBNP, and Troponin T), echocardiographic parameters, and cardiac MRI (cMR) findings were recorded at baseline. Follow-up data was available to a minimum of 32 months or until death. Follow-up included regular clinical assessments, echocardiography, laboratory testing, and additional imaging as clinically indicated. All patients provided written informed consent prior to participation. The study was conducted in accordance with the Declaration of Helsinki and reviewed and approved by the Vienna General Hospital, Medical University of Vienna Institutional Review Board (EK1557/2020) and requirement to obtain written informed consent was waived.

### ^11^C-PiB PET/CT imaging and analysis

Patients received a mean intravenous injection dose of 555 MBq (15mCi) of ^11^C-PiB. PET imaging was performed using a dedicated PET/CT scanner (Biograph Vision, Siemens, Germany). After whole-body low-dose CT from the base of the skull to the upper part of the femur, three-dimensional PET scans were conducted at 2 min per bed position 1 h after tracer injection. PET images were reconstructed on 128 × 128 matrices using an ordered subset expectation maximization method (2 iterations, 16 subsets) with a 3.5 mm Gaussian filter. For the quantitative analyses, we calculated the standardized uptake value maximum (SUVmax) and standardized uptake value peak (SUVpeak). VOI with a 2–3 cm diameter was placed over the region of highest uptake in the left myocardium. Similarly, the backgrounds were measured by VOIs placed over the adjacent vertebra and paraspinal muscle. Quantitative analysis involved calculating the myocardial SUV ratios based on adjacent vertebral and paraspinal muscle VOIs.

### DPD Scintigraphy and analysis

Patients received a mean intravenous injection of 740 MBq (20 mCi) of ^99m^Tc-DPD. Planar whole-body scintigraphy and single-photon emission computed tomography (SPECT) imaging were performed 2–3 h post-injection using a dual-head gamma camera (Symbia, Siemens, Germany). Whole-body images were acquired at a scan speed of 25 cm/min. A SPECT/CT of the chest was immediately performed in 180 configurations, step-and-shoot acquisition with body contour, 64 views, 20s per view, 256 × 256 matrix, and CT scans (130 kV, 35 mAs) were obtained for attenuation correction. The SPECT/CT images were reconstructed using the xSPECT/X-QUANT algorithm. For the quantification of cardiac uptake, an automated deep learning system for the extracting of a scintigraphy-based cardiac amyloid marker (CAM) was employed. The CAM is established via a multi-step process. First, a deep learning-based thorax detection is performed. Next, a histogram standardization is performed before the resulting image is used as input for a DenseNet121 convolutional neural network. The resulting probability-like score is the CAM. A CAM above 0.5 is representative of Perugini grade of 2 or 3 [7]. The CAM was previously validated for its high performance in detecting CA, particularly ATTR [8]. For immediate clinical application of the findings, a Perugini grade of ≥ 2 can be used alternatively to the CAM.

### Machine learning-based cardiac amyloidosis detection and subtyping

A logistic regression model with a one-versus-rest algorithm was employed to perform a multi-class classification differentiating between ATTR, AL, and nocardiac amyloidosis (non-CA). For the analysis, demographic features (Gender, BMI, DiagnosisAge), NYHA stage, blood parameters (proBNP, Troponin T, Free Kappa Chain, Free Lambda Chain), CAM, PIB PET/CT imaging parameters (Heart SUVmax, Bone SUVmax, Muscle SUVmax, Heart SUVpeak, Bone SUVpeak, Muscle SUVpeak, Heart to Bone SUVmax, Heart to Bone SUVpeak, Heart to Muscle SUVmax, Heart to Muscle SUVpeak, Bone to Muscle SUVmax, Bone to Muscle SUVpeak), echocardiography parameters (left ventricle [LV], right ventricle [RV], left atrium [LA], right atrium [RA], aorta ascendens [AoAsc], interventricular septum [IVS]), and cMR parameters (left atrium [LA], right atrium [RA], left ventricular ejection fraction [LVEF], right ventricular ejection fraction [RVEF], left ventricular end-diastolic volume [LVEDV], left ventricular end-systolic volume [LVESV], left ventricular end-diastolic diameter [LVEDD], right ventricular end-diastolic diameter [RVEDD], left ventricular stroke volume [LVSV], LA area, RA area) were employed, resulting in a total of 38 features. The synthetic minority oversampling technique (SMOTE) [9] was used to balance the minority class in the training sets. Feature selection was performed using the minimum redundancy maximum relevance (mRMR) [10] algorithm. To ensure robust performance estimation, we conducted 100-fold stratified Monte Carlo cross-validation, with 80% of samples randomly assigned to the training set and 20% to the test set. To gain insights into the predictive importance and directionality of individual features, we employed SHapley Additive exPlanations (SHAP) [11].

### Rationale-based cardiac amyloidosis detection and subtyping

Since applying machine learning models in clinical practice can be challenging, we further developed a rationale-based model grounded in prior clinical knowledge. In the rationale-based model, it was assumed that high cardiac ^99m^Tc-DPD scintigraphy uptake (Perugini grade 2 or 3) indicates ATTR while ^11^C-PiB indicates AL. For patients with a combined^99m^Tc-DPD and ^11^C-PiB uptake, the presence of ATTR was assumed. As cut-off for the detection of AL on ^11^C-PiB images, we employed the median for heart-to-muscle SUV_max_ (> 1.74). Evaluation of the model was performed using the same diagnostic performance metrics as for the machine learning model on the entire dataset.

### Survival prediction

A machine learning model was established to predict overall survival. For this purpose, a random forest model with 100 decision trees was trained in a 100-fold stratified Monte Carlo cross-validation scheme. Class balancing was performed using SMOTE, feature selection using mRMR and feature importance ranking using SHAP. Based on the machine learning model prediction of whether a patient will survive more than 5 years, the patients were divided into two groups. The groups were subsequently analyzed using conservative statistical methods, including the Logrank Test and Cox proportional hazard models.

### Statistical analysis

Sensitivity (SNS), specificity (SPC), positive predictive value (PPV), and negative predictive value (NPV) of ^11^C-PiB PET, ^99m^Tc-DPD scintigraphy, and their combination were calculated using biopsy results or genetic testing as the gold standard. The performance of machine learning and rationale-based models was additionally evaluated using the area under the receiver operating characteristic curve (AUC), accuracy (ACC), and balanced accuracy (BAC). For multi-class classifications, diagnostic metrics were computed via a one-versus-rest approach. 95% confidence intervals (CI) for machine learning performances were calculated over all cross-validation folds and using 10,000 bootstrap samples for rationale-based models. Univariate differences between ATTR, AL, and non-CA groups were assessed using the Kruskal-Wallis test. Time-to-event analysis was performed using Kaplan-Meier estimators and the Logrank Test was used to evaluate the prognostic significance of imaging findings. Patients were stratified based on their median value for the individual parameters. Cox proportional hazard models were used to assess the impact of the machine learning model on survival. Cox models were adjusted for age and sex. Continuous data are displayed as mean and standard deviation (SD) or as median with interquartile ranges (IQR). Categorical variables are shown as numbers with percentages. Statistical analyses were performed using Python (Version 3.9.5), SPSS (Version 25.0), and R (Version 3.6.1). A *p* value of < 0.05 was considered statistically significant. Bonferroni correction was applied for multiple testing correction where applicable.

### Diagnostic performance of individual parameters

In addition to the univariate statistical analysis, a diagnostic performance evaluation of the significant parameters was carried out individually for each parameter. For this purpose, parameters where either stratified based on the median or in the case of the CAM at 0.5 (Perugini grade ≥ 2). Subsequently, diagnostic performance metrics were calculated to assess the ability to the parameters to predict either ATTR or AL amyloidosis.

## Results

### Patients

**Fig. 1:**
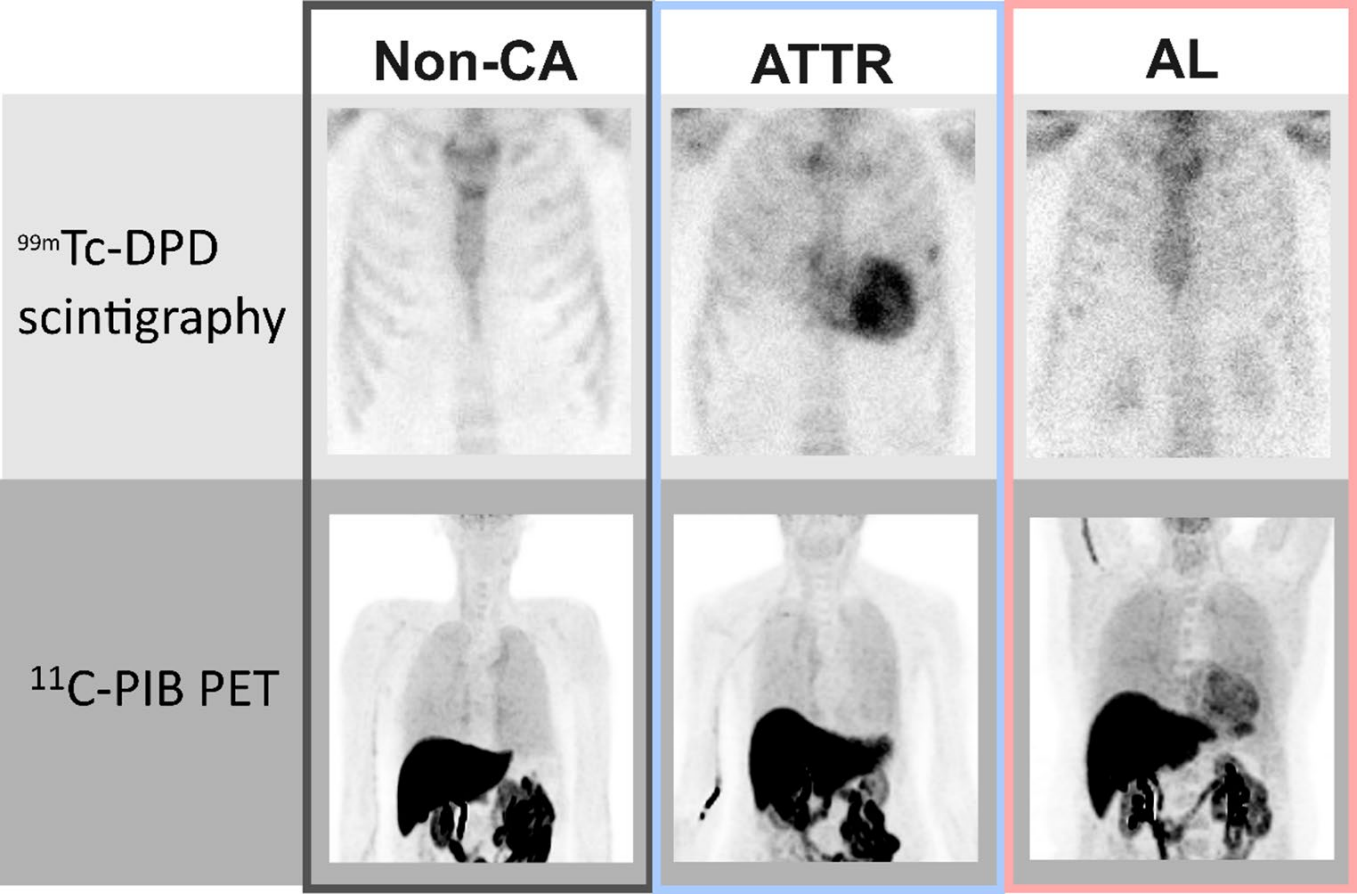
Example images for patients with ATTR, AL, and Non-cardiac amyloidosis. The top row represents scintigraphy images acquired with ^99m^Tc-DPD, the lower row represents PET images acquired with ^11^C-PiB. The left column shows a patient without cardiac amyloidosis (Non-CA), the middle column shows a patient with ATTR and the right column shows a patient with AL

Overall, 50 patients with available ^11^C-PiB PET, ^99m^Tc-DPD, and biopsy or genetic data for definite CA diagnosis were eligible for this study and were retrospectively enrolled. Patient characteristics stratified with respect to the presence of either ATTR, AL, or Non-CA are shown in Table 1. Of the 50 patients enrolled, 15 (30%) were confirmed to have ATTR, 12 (24%) had AL, and 23 (46%) did not have CA based on either genetic testing or biopsy. None of the patients had both, ATTR and AL. Example images are shown in Fig. 1. In the study cohort, men were more common compared to females with 34 (68%) male versus 16 (32%) female patients. The mean age was 71 ± 10years, with non-CA patients beingyounger (67 ± 11 years) than ATTR patients (76 ± 7 years), and AL patients (71 ± 11 years). The mean follow-up time was 65 months, after which 24 (48%) had died.

**Table 1:** Patient characteristics

Characteristic	Overall(*n* = 50)	AL(*n* = 12)	ATTR(*n* = 15)	Non-CA (*n* = 23)
Female	16 (32%)	4 (33%)	3 (20%)	9 (39%)
Age (y)	71 ± 10	71 ± 11	76 ± 7	67 ± 11
Body mass index (kg/m^2^)	27.4 ± 5.0	26.8 ± 3.6	26.8 ± 4.3	28.1 ± 6.0
NYHA_score				
I	5 (10%)	0 (0%)	1 (7%)	4 (17%)
II	28 (56%)	7 (58%)	10 (67%)	11 (48%)
III	17 (34%)	5 (42%)	4 (26%)	8 (35%)
NT-proBNP (pg/mL)	1894 (791–4436, IQR = 3644)	2031 (1438–5875, IQR = 4437)	2311 (1020–4406, IQR = 3386)	835 (103–4217, IQR = 4114)
TroponinT (ng/L)	23.5 (15.8–42.5, IQR = 26.8)	54.5 (27.8–90, IQR = 62.3)	39 (15–44, IQR = 29)	18 (7–22, IQR = 15)
k (mg/L)	21.5 (15.6–46.5, IQR = 31)	16.5 (8.6–37.6, IQR = 29)	19.8 (16.6–26.9, IQR = 10.3)	29.4 (17.4–69.3, IQR = 51.9)
λ (mg/L)	41.5 (27.2–81.9, IQR = 54.7)	120.5 (37.6-381.8, IQR = 344)	33.2 (21.3–47, IQR = 25.7)	38 (27.8–68.8, IQR = 41)
SPECT				
CAM	0.30 ± 0.46	0.09 ± 0.28	0.93 ± 0.26	0.00 ± 0.00
PET				
Heart SUVmax	3.06 ± 2.82	6.14 ± 4.41	2.65 ± 0.99	1.71 ± 0.46
Heart SUVpeak	2.10 ± 2.06	4.27 ± 3.33	1.69 ± 0.71	1.24 ± 0.34
Heart to bone SUVmax	1.81 ± 3.11	4.37 ± 5.77	1.17 ± 0.43	0.90 ± 0.20
Heart to muscle SUVmax	2.49 ± 2.10	4.59 ± 3.03	2.28 ± 1.64	1.54 ± 0.39
Heart to bone SUVpeak	1.94 ± 3.09	4.42 ± 5.72	1.27 ± 0.61	1.09 ± 0.46
Heart to muscle SUVpeak	2.61 ± 2.15	4.59 ± 3.08	2.30 ± 1.91	1.78 ± 0.57
Cardiac MRI				
LA (mm)	66 ± 7	66 ± 7	68 ± 8	64 ± 7
RA (mm)	64 ± 8	62 ± 6	69 ± 7	65 ± 11
LVEF (%)	58 ± 11	64 ± 9	51 ± 8	59 ± 13
RVEF (%)	51 ± 10	53 ± 8	47 ± 10	54 ± 12
LVEDV (mL)	152 ± 38	129 ± 30	169 ± 29	154 ± 46
LVESV (mL)	67 ± 28	46 ± 18	86 ± 26	64 ± 30
LVEDD (mm)	47 ± 7	44 ± 5	48 ± 6	48 ± 9
RVEDD (mm)	41 ± 5	40 ± 4	42 ± 6	42 ± 7
LVSV (mL)	86 ± 23	83 ± 23	84 ± 20	89 ± 31
ECV (%)	42 ± 16	49 ± 17	51 ± 13	29 ± 5
iLVM (g/m^2^)	95 ± 32	79 ± 22	116 ± 30	87 ± 33
Echocardiography				
LV (mm)	43 ± 8	41 ± 7	38 ± 9	46 ± 7
RV (mm)	35 ± 6	30 ± 5	35 ± 6	37 ± 6
LA (mm)	62 ± 8	62 ± 9	63 ± 8	61 ± 9
RA (mm)	60 ± 8	58 ± 5	62 ± 8	59 ± 10
IVS (mm)	17 ± 4	17 ± 3	21 ± 4	15 ± 4

AL = light chain amyloidosis; ATTR = transthyretin amyloidosis; Non-CA = no Cardiac amyloidosis; NYHA = New York Heart Association; NT-proBNP = N-terminal pro-B-type natriuretic peptide; k = Free Kappa Light Chain; λ = Free Lambda Light Chain; SPECT = single photon emission computed tomography; CAM = cardiac amyloid marker; PET = positron emission computed tomography; SUV = standardized uptake value; LA = left atrium; RA = right atrium; LVEF = left ventricular ejection fraction; RVEF = right ventricular ejection fraction; LVEDV = Left ventricular end-diastolic volume; LVESV = left ventricular end-systolic volume; LVEDD = left ventricular end-diastolic diameter; RVEDD = right ventricular end-diastolic diameter; LVSV = left ventricular stroke volume; ECV = extracellular volume; iLVM = indexed left ventricular mass; LV = left ventricle; RV = right ventricle; IVS = interventricular septum

### Cardiac amyloidosis subtyping of individual markers

**Fig. 2:**
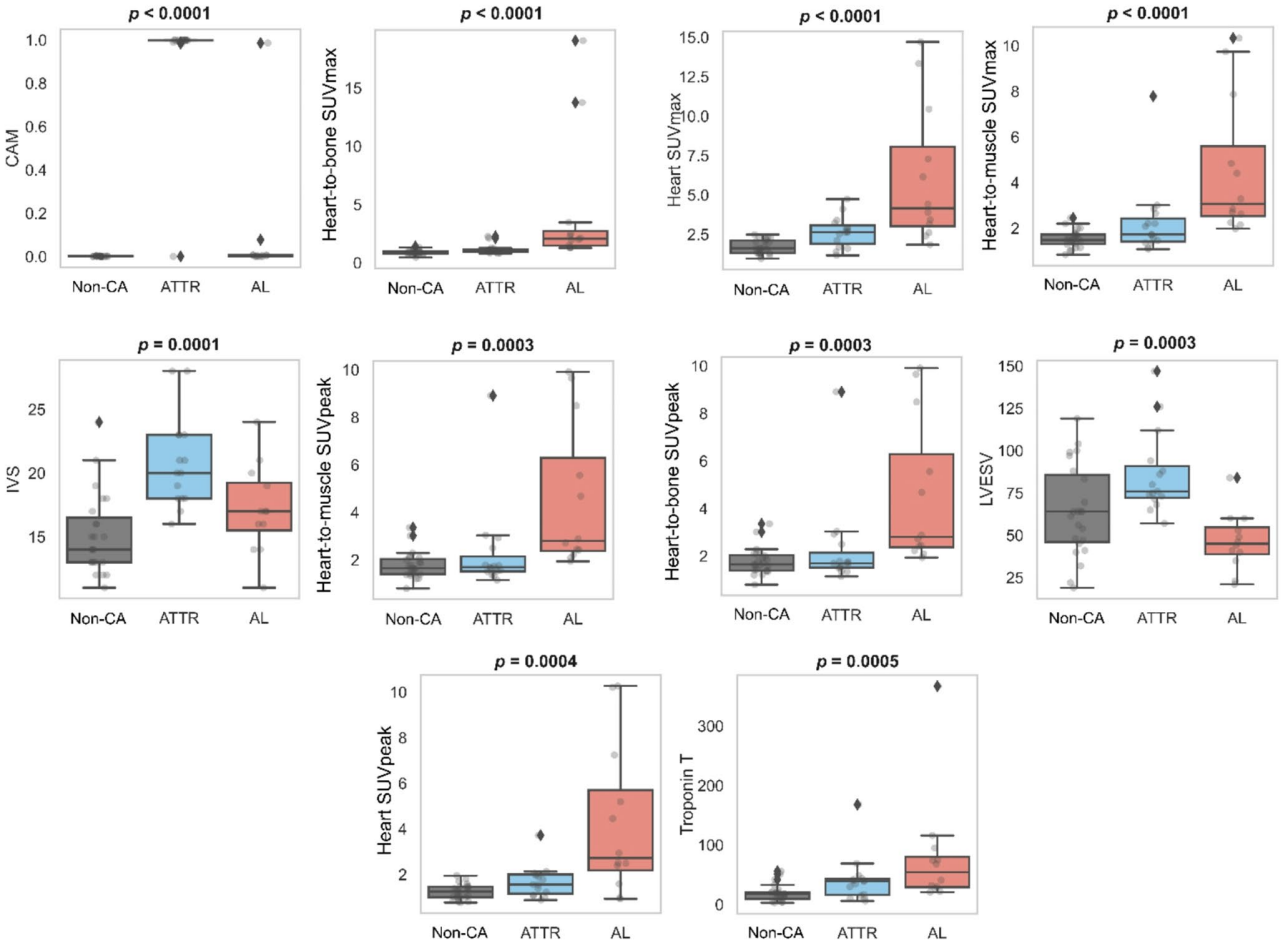
Distribution of the ten significant markers for the univariate differentiation of ATTR, AL, and Non-CA. Markers are ordered by *p* value from left to right and from top to bottom. Despite the strong ability of some markers to differentiate at least one group from the others, none of the markers is able to accurately differentiate all three groups

All 38 parameters were assessed for their ability to differentiate patients with ATTR, AL, and patients without CA. In ten parameters, there was a significant difference for at least one of the three groups with a Bonferroni corrected significance level of *p* < 0.0013. The ten parameters included one blood parameter, one echocardiography parameter, one scintigraphy parameter, and six PET/CT derived parameters: CAM (*p* < 0.0001), heart-to-bone SUVmax (*p* < 0.0001), heart SUVmax (*p* < 0.0001), heart-to-muscle SUVmax (*p* < 0.0001), IVS (*p* = 0.0001), heart-to-muscle SUVpeak (*p* = 0.0003), heart-to-bone SUVpeak (*p* = 0.0003), LVESV (*p* = 0.0003), heart SUVpeak (*p* = 0.0004) and troponin T (*p* = 0.0005). Distributions of the significantly associated markers are shown in Fig. 2. The CAM allowed for an almost perfect stratification of ATTR patients at a cut-off of 0.5 with only one false negative and one false positive, which was positive for AL-CA based on biopsy. For AL, heart-to-bone SUVmax provided the best detection with only 4 false positives (2 actual negatives and 2 ATTR) when using an optimal cut-off of 1.27. However, although some markers strongly differentiated at least one group (ATTR, AL, Non-CA) from the others, none of the markers wereable to accurately distinguish all three groups. Despite the markers’ diagnostic ability, most were not associated with overall mortality except for heart SUVmax (*p =* 0.01), IVS (*p* = 0.03), and Troponin T (*p* = 0.005).

In the diagnostic performance assessment of individual parameters, CAM/Perugini grade yielded the best performance for ATTR amyloidosis (AUC 0.95) followed by LVESV (AUC 0.84) and IVS (0.80). For the prediction of AL amyloidosis, the highest performance was achieved by the heart-to-bone SUVmax (AUC 0.84), followed by heart-to-muscle SUVmax, heart-to-muscle SUVpeak and heart-to-bone SUVpeak (all AUC 0.83). A comprehensive listing of performances of individual parameters is shown in Supplementary Table S1 and S2 for ATTR and AL amyloidosis respectively.

### Machine learning-based cardiac amyloidosis detection and subtyping

**Fig. 3:**
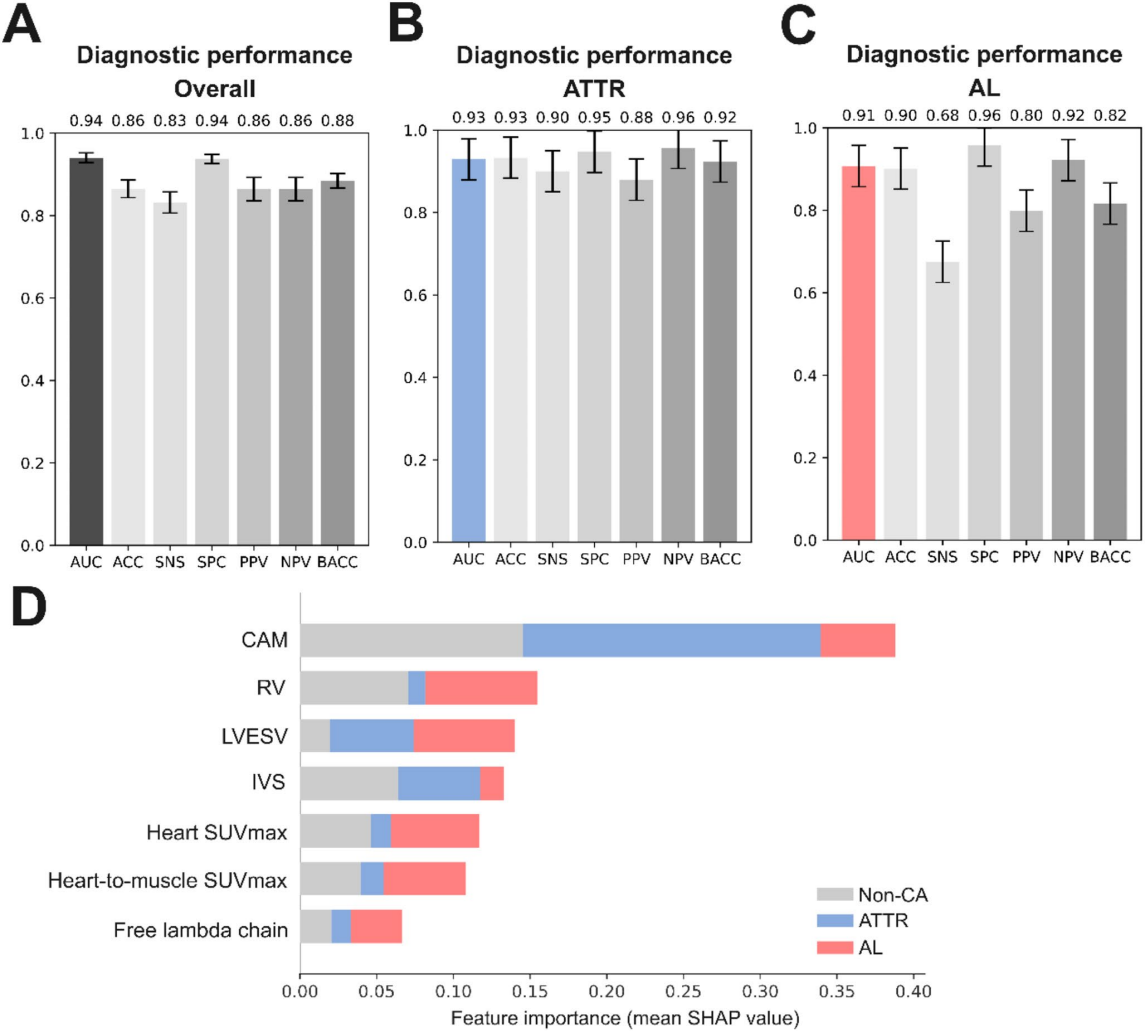
Results for the cardiac amyloidosis detection and subtyping model. **A** multi-class classification performance for the differentiation of ATTR, AL, and Non-Cardiac amyloidosis. **B** Diagnostic performance for ATTR. **C** Diagnostic performance for AL. **D** Most predictive parameters in the machine learning model

To potentially allow for the common stratification into Non-CA, ATTR, and AL, we established a multivariate machine learning model using all 38 predictor variables. In the 100-fold Monte Carlo cross-validation, the developed logistic regression model achieved an AUC of 0.94 (95% CI 0.93–0.95) for the differentiation into the three groups. The machine learning approach further demonstrated a sensitivity of 0.83 (95% CI 0.80–0.86), specificity of 0.94 (95% CI 0.93–0.95), positive predictive value of 0.86 (95% CI 0.83–0.89), negative predictive value of 0.86 (95% CI 0.83–0.89), balanced accuracy of 0.88 (95% CI 0.86–0.90), and overall accuracy of 0.87 (95% CI 0.85–0.89) as shown in Fig. 3. ATTR and AL-specific performances were computed with a one-versus-rest approach based on the predictions of the multi-class model. The model demonstrated a similar overall diagnostic performance for ATTR and AL (AUC 0.93 versus AUC 0.91). However, the sensitivity of the model for the detection of ATTR was substantially better compared to AL (SNS 0.90 versus SNS 0.68). The specificity was high for both subtypes with a SPC 0.95 for ATTR and a SPC of 0.96 for AL.

### Rationale-based cardiac amyloidosis detection and subtyping

**Fig. 4:**
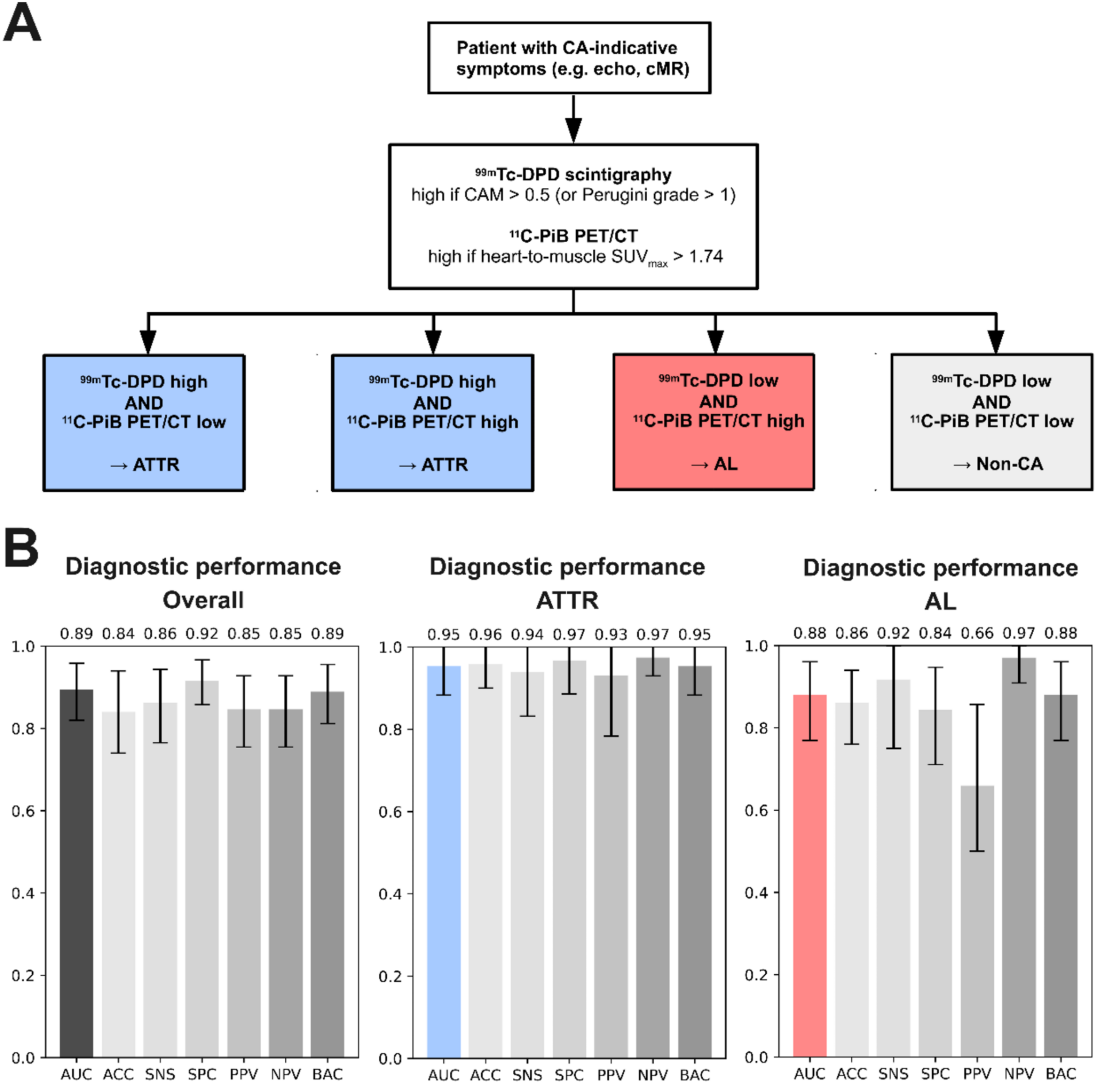
The rationale-based model provides an easy-to-follow structure that can be implemented in clinical routine. Perugini grade ≥ 2 can be used as an alternative to CAM > 0.5 to enhance immediate clinical applicability. **A** Rationale-based model always assuming ATTR if ^99m^Tc-DPD is high and AL if ^11^C-PiB PET shows high uptake while ^99m^Tc-DPD shows low uptake. **B** Diagnostic performance resulting from the implementation of the proposed rationale-based model

The rationale-based CA model proposes a clinical algorithm for the imaging-based diagnostic workup of patients with suspected cardiac amyloidosis (Fig. 4). The results indicate strong diagnostic performance for the detection of both, ATTR (AUC 0.95 [95% CI 0.88-1.00]) and AL (AUC 0.88 [95% CI 0.770–0.961]) with comparable sensitivities for AL (SNS 0.92 [95% CI 0.750 to 1.000]) and ATTR (SNS 0.94 [95% CI 0.750-1.000]). Overall, there were 7/50(13%) patients incorrectly detected by the proposed model, with the vast majority (5/7, 71%) caused by false positive predictions of AL in patients without CA, indicating the necessity for increased caution when AL amyloidosis is detected using this model. All performance metrics for ATTR, AL, and the overall prediction are shown in Fig. 4.

### Survival prediction

**Fig. 5:**
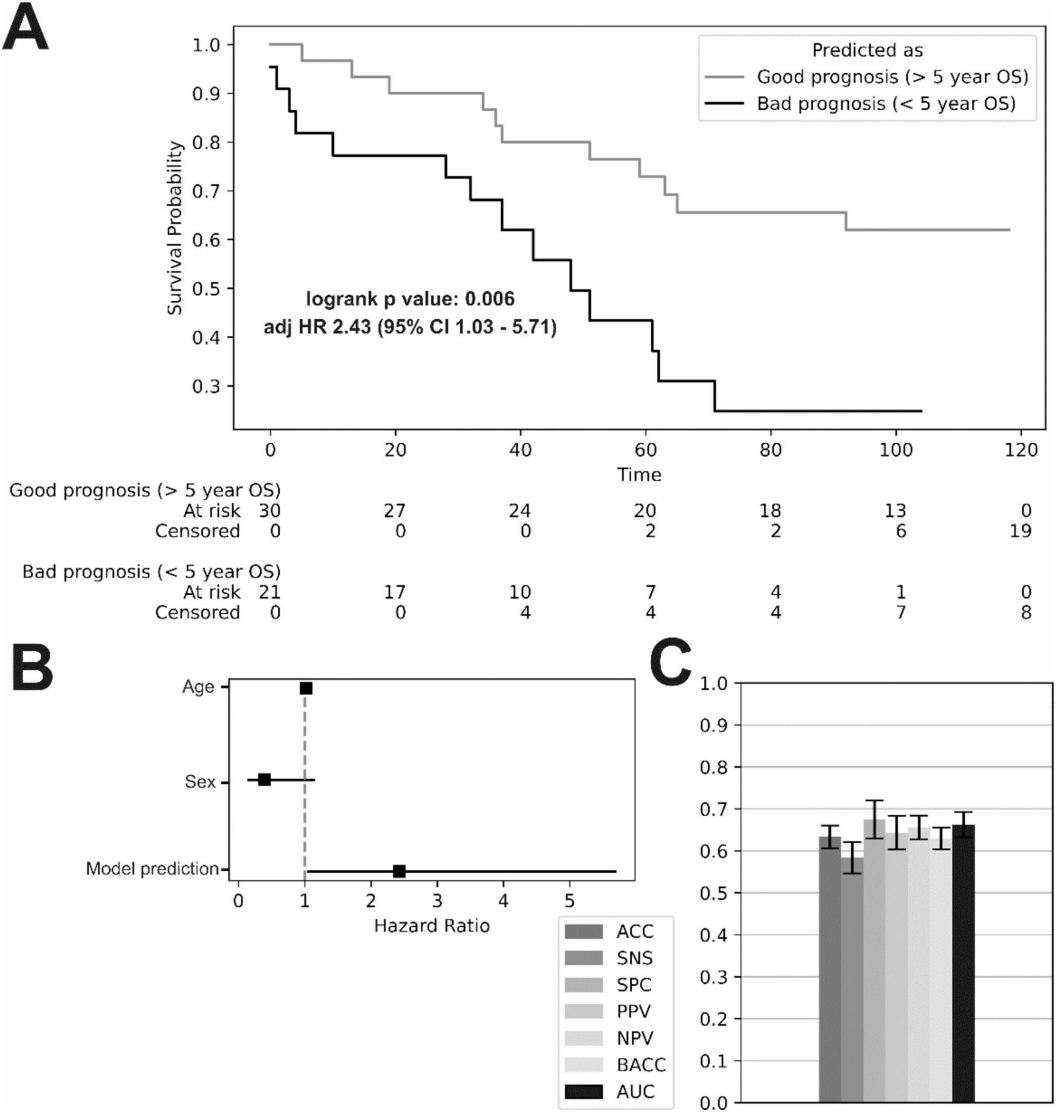
Results of the survival prediction model **A** Kaplan Meier estimator of the 5-year survival model. **B** Hazard ratios of parameters in the adjusted Cox proportional hazards model. **C** Machine learning performance of the 5-year survival model

The 5-year survival prediction model reached an AUC of 0.66 (95% CI 0.63–0.69). However, despite the mediocre predictive performance, stratification of the patient population based on whether the model predicted survival of more than 5 years was significantly associated with overall mortality (*p* value 0.006) and remained significant after adjusting for age and sex (adj HR 2.43 [95% CI 1.03–5.71). Based on SHAP feature importance, the most prognostic parameters included Troponin T, age, heart-to-muscle ratio SUVpeak, NT-proBNP, LVEDD, heart-to-muscle ratio SUVmax, and sex. The results of the survival prediction are shown in Fig. 5.

## Discussion

The differentiation between CA subtypes is critical because they have distinct prognostic and therapeutic implications. In this retrospective study, we analyzed 50 patients with clinically suspected cardiac amyloidosis. These patients underwent both ^11^C-PiB PET/CT and ^99m^Tc-DPD scintigraphy, and their imaging results were compared against biopsy or genetic testing, which served as the gold standard for diagnosis. We assessed the diagnostic accuracy and subtyping capabilities of cardiac amyloidosis with a focus on image-derived parameters from both scans and demonstrated their prognostic value.

The study population included patients with confirmed ATTR-CA, AL-CA, or Non CA, providing a diverse cohort for evaluating the diagnostic performance of the imaging techniques. The methodology employed in this study was rigorous, incorporating both qualitative and quantitative analyses of the imaging data. For ^11^C-PiB PET/CT, quantitative parameters such as SUV_max_ and SUV_peak_ were calculated by placing volumes of interest (VOIs) over regions of high uptake in the left myocardium. These values were then normalized against background VOIs placed over adjacent vertebrae and paraspinal muscle. Similarly, ^99m^Tc-DPD scintigraphy involved the calculation of the CAM, which was derived using an automated deep learning system. The CAM was previously validated for its high performance in detecting CA, particularly ATTR. While the CAM has been shown to be more standardized avoid inter-rater variability, the visual Perugini grading can still be employed as an alternative, making particularly the rationale-based model simple to use in a clinical scenario. In addition to imaging, we also considered various clinical parameters, including demographic data, NYHA stage, laboratory results (e.g., serum-free light chains, NT-proBNP, Troponin T), echocardiographic parameters, and cardiac MRI findings. This comprehensive dataset allowed for the development of two predictive models: a machine learning-based model and a rationale-based model.

We showed that quantitative nuclear medicine imaging parameters enable the detection of individual cardiac amyloidosis subtypes, however, they are not capable of detecting and subtyping simultaneously. Therefore, we proposed two predictive modeling approaches integrating multiple parameters to improve simultaneous detection and differentiation of subtypes. First, we developed a machine learning model integrating 29 imaging parameters from ^99m^Tc-DPD and ^11^C-PiB, echocardiography, and cardiac MRI alongside 9 non-invasive or minimally invasive parameters. The results indicated a strong diagnostic performance of the model, holding true for the overall prediction of cardiac amyloidosis as well as for the reliable differentiation into subtypes. Our results further suggested that prognostic machine learning models can be built incorporating both, imaging and non-imaging parameters. Despite the rapidly increasing number of routinely applied machine learning-driven procedures in clinics, particularly in cardiovascular imaging [12], the implementation of such systems is associated with substantial challenges and requires enormous efforts [13, 14]. As a consequence, we proposed a second “rationale-based” model based on prior clinical knowledge, which can be implemented as a simple diagnostic workflow in clinical practice. Despite the slight difference in overall performance (AUC 0.94 versus AUC 0.89), the rationale-based model demonstrated remarkable diagnostic ability. In addition, while the sensitivity of the machine learning approach was high for the detection of ATTR, AL detection was substantially lower compared with the rationale-based model (SNS 0.92 versus SNS 0.68). Due to its poor prognosis and often rapid progression as well as the availability of additional diagnostic methods, sensitivity is of particular importance for AL diagnosis. The rule-based model further convinces by its simplicity of the employed parameters. With only two parameters, one from ^99m^Tc-DPD and one from ^11^C-PiB, it was able to detect cardiac amyloidosis as well as differentiate between ATTR and AL subtypes. Owing to its low complexity, the rationale-based model can be easily integrated with additional clinical knowledge on a day-by-day basis, while the machine learning model only allows for limited interpretability.

The results of this study have to be interpreted cautiously due to some limitations. Firstly, Our study was retrospective in design and limited to static imaging data, whereas dynamic data could offer more valuable information in distinguishing ATTR from AL amyloidosis or non-amyloidosis patients. Secondly, While being larger than many other studies investigating AL detection and/or cardiac amyloidosis subtyping using nuclear medicine imaging [6, 15–19], the study population is small, which is particularly limiting for the machine learning approach. While substantial progress has been made in the field of generalizability of machine learning systems applied to medical imaging tasks [20, 21], external validation is still recommended to assess the exact performance of imaging-based machine learning models. A major limitation of both proposed models is their inability to detect the concurrent presence of multiple types of cardiac amyloidosis. While the probability of having both subtypes is not nill and rises with age, the simultaneous presence of both subtypes is considered rare [22–24]. Furthermore, rare CA subtypes not considered for the detection by the proposed models include amyloid A amyloidosis, apolipoprotein A1 amyloidosis, and isolated atrial amyloidosis. Lastly, multiple studies have suggested the possibility of detecting AL amyloidosis with tracers beyond ^11^C-PiB, including with ^18^F-Florbetaben [15, 16], ^18^F-Flutemetamol [18], and ^18^F-Florbetapir [19]. Albeit their similarity to ^11^C-PiB, the translatability of our findings to these tracers is subject to future research.

## Conclusion

While ^99m^Tc-DPD scintigraphy parameters can accurately detect ATTR-CA and ^11^C-PiB PET/CT parameters allow for the detection of AL-CA, none of the two enable the reliable differentiation of both subtypes. This study presented the development and validation of two approaches for the detection and subtyping of cardiac amyloidosis using ^99m^Tc-DPD scintigraphy and ^11^C-PiB PET/CT. While the machine learning approach allows for better diagnostic performance, the rationale-based approach allows for the simple integration into the clinical workflow to detect both, ATTR and AL cardiac amyloidosis.

## Electronic supplementary material

Below is the link to the electronic supplementary material.

Supplementary Material 1

## Data Availability

The data underlying this article will be shared on reasonable request to the corresponding author.
