# Supplementary material for: Enhanced diagnostic and prognostic assessment of cardiac amyloidosis using combined 11C-PiB PET/CT and 99mTc-DPD scintigraphy
Source: Eur J Nucl Med Mol Imaging. 2025 Feb 28;52(9):3321–32. doi: 10.1007/s00259-025-07157-7 (PMC12222433; doi:10.1007/s00259-025-07157-7)
Supplement: Supplementary file 1 — Supplementary Material 1 [file 259_2025_7157_MOESM1_ESM.docx]

**Supplemental file**

**Enhanced Diagnostic and Prognostic Assessment of Cardiac Amyloidosis Using Combined ^11^C-PiB PET/CT and ^99m^Tc-DPD Scintigraphy**

Zhihui Hong^1,2^*, Clemens P. Spielvogel^2^*, Song Xue^2^, Raffaella Calabretta^2^, Zewen Jiang^2^, Josef Yu^2^, Kilian Kluge^2^, David Haberl^2,4^, Christian Nitsche^3^, Stefan Grünert^2^, Marcus Hacker^2^ and Xiang Li^2,5#^

^1^Department of Nuclear Medicine, The Second Affiliated Hospital of Soochow University, Suzhou 215002, China

^2^Division of Nuclear Medicine, Department of Biomedical Imaging and Image-guided Therapy, Vienna General Hospital, Medical University of Vienna, Währinger Gürtel 18-20, Floor 3L, 1090 Vienna, Austria

^3^Division of Cardiology, Department of Internal Medicine II, Medical University of Vienna, Vienna, Austria

^4^Christian Doppler Laboratory for Applied Metabolomics, Medical University of Vienna, Vienna, Austria

^5^Department of Nuclear Medicine, Beijing Chest Hospital, Capital Medical University. Beijing, China.

* First authors with shared contribution

Li X. and Hacker M. Shared the senior authorship

*Correspondence to:*

Prof. Dr. Xiang Li

Division of Nuclear Medicine, Department of Biomedical Imaging and Image-guided Therapy, Medical University of Vienna, Vienna, Austria

Xiang.li@meduniwien.ac.at

**Table S1**. Single parameter performance for ATTR amyloidosis prediction.

|  | **ACC** | **SNS** | **SPC** | **PPV** | **NPV** | **BACC** | **AUC** |
| --- | --- | --- | --- | --- | --- | --- | --- |
| CAM / Perugini grade≥2 | 0.96 | 0.93 | 0.97 | 0.93 | 0.97 | 0.95 | 0.95 |
| Heart-to-bone SUVmax | 0.54 | 0.53 | 0.54 | 0.33 | 0.73 | 0.54 | 0.54 |
| Heart SUVmax | 0.60 | 0.67 | 0.57 | 0.40 | 0.80 | 0.62 | 0.62 |
| Heart-to-muscle SUVmax | 0.48 | 0.47 | 0.49 | 0.28 | 0.68 | 0.48 | 0.48 |
| IVS | 0.78 | 0.87 | 0.74 | 0.59 | 0.93 | 0.80 | 0.80 |
| Heart-to-muscle SUVpeak | 0.36 | 0.27 | 0.40 | 0.16 | 0.56 | 0.33 | 0.33 |
| Heart-to-bone SUVpeak | 0.36 | 0.27 | 0.40 | 0.16 | 0.56 | 0.33 | 0.33 |
| LVESV | 0.80 | 0.93 | 0.74 | 0.61 | 0.96 | 0.84 | 0.84 |
| Heart SUVpeak | 0.58 | 0.60 | 0.57 | 0.38 | 0.77 | 0.59 | 0.59 |
| Troponin T | 0.60 | 0.67 | 0.57 | 0.40 | 0.80 | 0.62 | 0.62 |

**Table S2**. Single parameter performance for AL amyloidosis prediction.

|  | ACC | SNS | SPC | PPV | NPV | BACC | AUC |
| --- | --- | --- | --- | --- | --- | --- | --- |
| CAM / Perugini grade≥2 | 0.48 | 0.21 | 0.56 | 0.23 | 0.23 | 0.38 | 0.64 |
| Heart-to-bone SUVmax | 0.76 | 1.00 | 0.68 | 0.50 | 1.00 | 0.84 | 0.84 |
| Heart SUVmax | 0.70 | 0.92 | 0.63 | 0.44 | 0.96 | 0.77 | 0.77 |
| Heart-to-muscle SUVmax | 0.74 | 1.00 | 0.66 | 0.48 | 1.00 | 0.83 | 0.83 |
| IVS | 0.48 | 0.33 | 0.53 | 0.18 | 0.71 | 0.43 | 0.43 |
| Heart-to-muscle SUVpeak | 0.74 | 1.00 | 0.66 | 0.48 | 1.00 | 0.83 | 0.83 |
| Heart-to-bone SUVpeak | 0.74 | 1.00 | 0.66 | 0.48 | 1.00 | 0.83 | 0.83 |
| LVESV | 0.34 | 0.08 | 0.42 | 0.04 | 0.59 | 0.25 | 0.25 |
| Heart SUVpeak | 0.68 | 0.83 | 0.63 | 0.42 | 0.92 | 0.73 | 0.73 |
| Troponin T | 0.66 | 0.83 | 0.61 | 0.40 | 0.92 | 0.72 | 0.72 |
